# 2-Chloro-*N*-(4-fluoro­phen­yl)acetamide

**DOI:** 10.1107/S1600536808016152

**Published:** 2008-06-07

**Authors:** Si-shun Kang, Hai-su Zeng, Hai-lin Li, Hai-bo Wang

**Affiliations:** a College of Science, Nanjing University of Technology, Xinmofan Road No. 5 Nanjing, Nanjing 210009, People’s Republic of China

## Abstract

In the title compound, C_8_H_7_ClFNO, an intra­molecular C—H⋯O hydrogen bond forms a six-membered ring. In the crystal structure, mol­ecules are linked by inter­molecular N—H⋯O hydrogen bonds, forming infinite chains along the *c* axis.

## Related literature

For related compounds, see: Wen *et al.* (2006 ▶); Zhang *et al.* (2006 ▶). For reference structural data, see: Allen *et al.* (1987 ▶).
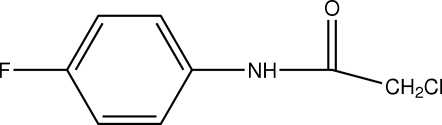


## Experimental

### 

#### Crystal data

C_8_H_7_ClFNO
*M* *_r_* = 187.60
Monoclinic, 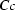

*a* = 4.7410 (9) Å
*b* = 20.062 (4) Å
*c* = 8.9860 (18) Å
β = 99.60 (3)°
*V* = 842.7 (3) Å^3^
*Z* = 4
Mo *K*α radiation
μ = 0.42 mm^−1^
*T* = 293 (2) K
0.30 × 0.20 × 0.05 mm

#### Data collection

Enraf–Nonius CAD-4 diffractometer
Absorption correction: ψ scan (North *et al.*, 1968 ▶) *T*_min_ = 0.885, *T*_max_ = 0.980
974 measured reflections
861 independent reflections
610 reflections with *I* > 2σ(*I*)
*R*_int_ = 0.015
3 standard reflections every 200 reflections intensity decay: none

#### Refinement

*R*[*F* ^2^ > 2σ(*F* ^2^)] = 0.046
*wR*(*F* ^2^) = 0.126
*S* = 1.00
861 reflections
103 parameters
H-atom parameters constrained
Δρ_max_ = 0.16 e Å^−3^
Δρ_min_ = −0.20 e Å^−3^
Absolute structure: Flack (1983 ▶), 92 Friedel pairs
Flack parameter: 0.18 (17)

### 

Data collection: *CAD-4 Software* (Enraf–Nonius, 1989 ▶); cell refinement: *CAD-4 Software*; data reduction: *XCAD4* (Harms & Wocadlo, 1995 ▶); program(s) used to solve structure: *SHELXS97* (Sheldrick, 2008 ▶); program(s) used to refine structure: *SHELXL97* (Sheldrick, 2008 ▶); molecular graphics: *SHELXTL* (Sheldrick, 2008 ▶); software used to prepare material for publication: *SHELXL97*.

## Supplementary Material

Crystal structure: contains datablocks global, I. DOI: 10.1107/S1600536808016152/hb2738sup1.cif

Structure factors: contains datablocks I. DOI: 10.1107/S1600536808016152/hb2738Isup2.hkl

Additional supplementary materials:  crystallographic information; 3D view; checkCIF report

## Figures and Tables

**Table 1:** Hydrogen-bond geometry (Å, °)

*D*—H⋯*A*	*D*—H	H⋯*A*	*D*⋯*A*	*D*—H⋯*A*
C3—H3*A*⋯O	0.93	2.36	2.925 (8)	119
N—H1⋯O^i^	0.86	2.02	2.853 (6)	164

Symmetry code: (i) 
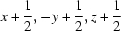
.

**Fig. 1.**
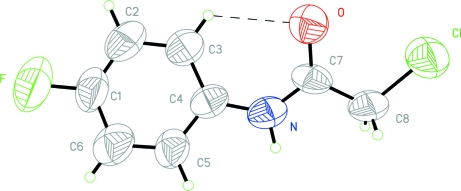
The molecular structure of (I) with displacement ellipsoids for the non-hydrogen atoms drawn at the 50% probability level. The intramolecular hydrogen bond is shown as a dashed line.

## supplementary crystallographic information

### Comment

N-(substituted phenyl)-2-chloroacetamides are important intermediates in organic synthesis. They can be used in the synthesis of many derivatives such as (quinolin-8-yloxy) acetamide (Zhang *et al.*, 2006) and 2,5-piperazinedione (Wen *et al.*, 2006). In our studies in this area, the title compound,(I), was synthesized and structurally characterised.

The bond lengths and angles in (I) are within normal ranges (Allen *et al.*, 1987).  An intramolecular C—H···O interaction occurs (Fig. 1) and an intermolecular N—H···O hydrogen bond helps to establish the packing (Table 1).

### Experimental

Chloroacetyl chloride (0.05 mol) was added to a solution of 4-nitrophenylamine (0.05 mol) and triethylamine (0.05 mol) in toluene (50 ml) over a period of 30 min, with cooling in an ice bath, and then the mixture was stirred at room remperature for 4 h. After separation of the triethylamine hydrochloride by filtration, the organic phase was washed three times with water. The toluene layer was removed and evaporated. Pink blocks of (I) were obtained by slow evaporation of a chloroform solution over a period of 7 d.

### Refinement

The H atoms were positioned geometrically (N—H = 0.86 Å, C—H = 0.93-0.97Å) and refined as riding with *U*_iso_(H) = *xU*_eq_(carrier).

### Crystal data

**Table:**

C_8_H_7_ClFNO	*F*_000_ = 384
*M**_r_* = 187.60	*D*_x_ = 1.479 Mg m^−^^3^
Monoclinic, *C**c*	Mo *K*α radiation λ = 0.71073 Å
Hall symbol: C -2yc	Cell parameters from 25 reflections
*a* = 4.7410 (9) Å	θ = 8–12º
*b* = 20.062 (4) Å	µ = 0.42 mm^−^^1^
*c* = 8.9860 (18) Å	*T* = 293 (2) K
β = 99.60 (3)º	Block, pink
*V* = 842.7 (3) Å^3^	0.30 × 0.20 × 0.05 mm
*Z* = 4	

### Data collection

**Table:**

Enraf–Nonius CAD-4 diffractometer	*R*_int_ = 0.016
Radiation source: fine-focus sealed tube	θ_max_ = 25.2º
Monochromator: graphite	θ_min_ = 2.0º
*T* = 293(2) K	*h* = 0→5
ω/2θ scans	*k* = 0→24
Absorption correction: ψ scan(North *et al.*, 1968)	*l* = −10→10
*T*_min_ = 0.885, *T*_max_ = 0.980	3 standard reflections
974 measured reflections	every 200 reflections
861 independent reflections	intensity decay: none
610 reflections with *I* > 2σ(*I*)	

### Refinement

**Table:**

Refinement on *F*^2^	Hydrogen site location: inferred from neighbouring sites
Least-squares matrix: full	H-atom parameters constrained
*R*[*F*^2^ > 2σ(*F*^2^)] = 0.046	*w* = 1/[σ^2^(*F*_o_^2^) + (0.*P*)^2^ + 0.5*P*] where *P* = (*F*_o_^2^ + 2*F*_c_^2^)/3
*wR*(*F*^2^) = 0.126	(Δ/σ)_max_ < 0.001
*S* = 1.00	Δρ_max_ = 0.16 e Å^−^^3^
861 reflections	Δρ_min_ = −0.20 e Å^−^^3^
103 parameters	Extinction correction: none
Primary atom site location: structure-invariant direct methods	Absolute structure: Flack (1983), 92 Friedel pairs
Secondary atom site location: difference Fourier map	Flack parameter: 0.18 (17)

### Special details

**Table:**

Geometry. All e.s.d.'s (except the e.s.d. in the dihedral angle between two l.s. planes) are estimated using the full covariance matrix. The cell e.s.d.'s are taken into account individually in the estimation of e.s.d.'s in distances, angles and torsion angles; correlations between e.s.d.'s in cell parameters are only used when they are defined by crystal symmetry. An approximate (isotropic) treatment of cell e.s.d.'s is used for estimating e.s.d.'s involving l.s. planes.
Refinement. Refinement of *F*^2^ against ALL reflections. The weighted *R*-factor *wR* and goodness of fit *S* are based on *F*^2^, conventional *R*-factors *R* are based on *F*, with *F* set to zero for negative *F*^2^. The threshold expression of *F*^2^ > σ(*F*^2^) is used only for calculating *R*-factors(gt) *etc*. and is not relevant to the choice of reflections for refinement. *R*-factors based on *F*^2^ are statistically about twice as large as those based on *F*, and *R*- factors based on ALL data will be even larger.

### Fractional atomic coordinates and isotropic or equivalent isotropic displacement parameters (Å^2^)

**Table:**

	*x*	*y*	*z*	*U*_iso_*/*U*_eq_	
Cl	−0.4033 (4)	0.15199 (10)	0.5685 (2)	0.1096 (7)	
N	0.1497 (11)	0.2970 (2)	0.6731 (5)	0.0738 (14)	
H1	0.2033	0.2848	0.7653	0.089*	
O	−0.1268 (10)	0.2652 (2)	0.4535 (5)	0.084	
F	0.6949 (14)	0.5279 (2)	0.5602 (6)	0.147 (2)	
C1	0.5590 (19)	0.4687 (3)	0.5826 (8)	0.095 (2)	
C2	0.3302 (19)	0.4493 (4)	0.4775 (8)	0.097 (2)	
H2A	0.2684	0.4749	0.3920	0.116*	
C3	0.1935 (15)	0.3901 (3)	0.5030 (6)	0.0817 (18)	
H3A	0.0450	0.3743	0.4308	0.098*	
C4	0.2759 (13)	0.3546 (3)	0.6344 (6)	0.0707 (15)	
C5	0.5091 (15)	0.3787 (3)	0.7356 (7)	0.0798 (17)	
H5A	0.5715	0.3539	0.8224	0.096*	
C6	0.6503 (19)	0.4357 (4)	0.7157 (8)	0.099 (2)	
H6A	0.7995	0.4516	0.7874	0.119*	
C7	−0.0366 (13)	0.2576 (3)	0.5950 (5)	0.0710 (16)	
C8	−0.1284 (15)	0.1998 (3)	0.6748 (6)	0.089 (2)	
H8A	−0.1937	0.2153	0.7654	0.107*	
H8B	0.0358	0.1712	0.7058	0.107*	

### Atomic displacement parameters (Å^2^)

**Table:**

	*U*^11^	*U*^22^	*U*^33^	*U*^12^	*U*^13^	*U*^23^
Cl	0.1293 (15)	0.1275 (15)	0.0742 (9)	−0.0277 (13)	0.0237 (9)	−0.0087 (10)
N	0.089 (3)	0.082 (3)	0.051 (2)	0.010 (3)	0.013 (2)	0.004 (2)
O	0.084	0.084	0.084	0.000	0.014	0.000
F	0.213 (7)	0.120 (3)	0.121 (3)	−0.062 (4)	0.067 (4)	0.001 (3)
C1	0.117 (6)	0.091 (5)	0.086 (5)	−0.031 (5)	0.043 (5)	0.001 (4)
C2	0.121 (6)	0.103 (5)	0.074 (4)	0.000 (5)	0.039 (4)	0.017 (4)
C3	0.090 (4)	0.096 (5)	0.063 (3)	−0.006 (4)	0.025 (3)	0.002 (3)
C4	0.078 (4)	0.080 (4)	0.058 (3)	0.008 (3)	0.020 (3)	0.008 (3)
C5	0.095 (4)	0.083 (4)	0.067 (3)	−0.016 (4)	0.029 (3)	0.000 (3)
C6	0.115 (6)	0.116 (5)	0.074 (4)	−0.007 (5)	0.038 (4)	0.010 (4)
C7	0.072 (3)	0.102 (4)	0.039 (2)	−0.003 (3)	0.009 (2)	−0.011 (3)
C8	0.111 (5)	0.110 (5)	0.044 (3)	−0.017 (4)	0.005 (3)	0.013 (3)

### Geometric parameters (Å, °)

**Table:**

Cl—C8	1.765 (7)	C3—C4	1.379 (8)
N—C7	1.300 (7)	C3—H3A	0.9300
N—C4	1.373 (8)	C4—C5	1.396 (9)
N—H1	0.8600	C5—C6	1.353 (10)
O—C7	1.281 (6)	C5—H5A	0.9300
F—C1	1.381 (7)	C6—H6A	0.9300
C1—C2	1.371 (10)	C7—C8	1.467 (8)
C1—C6	1.372 (10)	C8—H8A	0.9700
C2—C3	1.390 (9)	C8—H8B	0.9700
C2—H2A	0.9300		
			
C7—N—C4	131.3 (5)	C6—C5—C4	124.2 (6)
C7—N—H1	114.3	C6—C5—H5A	117.9
C4—N—H1	114.3	C4—C5—H5A	117.9
C2—C1—C6	124.2 (7)	C5—C6—C1	115.6 (7)
C2—C1—F	118.6 (7)	C5—C6—H6A	122.2
C6—C1—F	117.0 (7)	C1—C6—H6A	122.2
C1—C2—C3	117.7 (6)	O—C7—N	123.2 (6)
C1—C2—H2A	121.1	O—C7—C8	120.1 (5)
C3—C2—H2A	121.1	N—C7—C8	116.5 (4)
C4—C3—C2	120.7 (6)	C7—C8—Cl	114.7 (4)
C4—C3—H3A	119.7	C7—C8—H8A	108.6
C2—C3—H3A	119.7	Cl—C8—H8A	108.6
N—C4—C3	125.4 (6)	C7—C8—H8B	108.6
N—C4—C5	117.3 (5)	Cl—C8—H8B	108.6
C3—C4—C5	117.3 (6)	H8A—C8—H8B	107.6
			
C6—C1—C2—C3	−4.3 (12)	C3—C4—C5—C6	2.8 (10)
F—C1—C2—C3	−179.1 (7)	C4—C5—C6—C1	−3.0 (11)
C1—C2—C3—C4	3.9 (11)	C2—C1—C6—C5	3.8 (12)
C7—N—C4—C3	11.2 (11)	F—C1—C6—C5	178.7 (7)
C7—N—C4—C5	−168.0 (7)	C4—N—C7—O	4.7 (11)
C2—C3—C4—N	177.7 (6)	C4—N—C7—C8	−179.3 (6)
C2—C3—C4—C5	−3.1 (10)	O—C7—C8—Cl	−9.0 (9)
N—C4—C5—C6	−177.9 (7)	N—C7—C8—Cl	174.8 (5)

### Hydrogen-bond geometry (Å, °)

**Table:**

*D*—H···*A*	*D*—H	H···*A*	*D*···*A*	*D*—H···*A*
C3—H3A···O	0.93	2.36	2.925 (8)	119
N—H1···O^i^	0.86	2.02	2.853 (6)	164

Symmetry codes: (i) *x*+1/2, −*y*+1/2, *z*+1/2.
